# Mobile thrombi in three chambers of the heart

**DOI:** 10.3402/jchimp.v3i3-4.22792

**Published:** 2013-12-17

**Authors:** Rachel Kunkler, David Scott Weisman, Ijaz Khan

**Affiliations:** 1 Ross University School of Medicine, North Brunswick, NJ, USA; 2 MedStar Good Samaritan Hospital, Baltimore, MD, USA; 3 Department of Cardiology, MedStar Good Samaritan Hospital, Baltimore, MD, USA

**Keywords:** mobile heart thrombi, patent foramen ovale, thromboemboli

## Abstract

A case report of pulmonary embolus complicated by mobile thrombus in three chambers of the heart with emphasis on treatment and management options.

A 74-year-old male with history of chronic stable angina, hypertension, peripheral vascular disease, and obesity presented to the office with the complaint of dizziness following a bout of gastroenteritis. On the day prior to presentation, after having been dehydrated and bedbound for several days due to his illness, he also experienced an episode of diaphoresis and near syncope at his home. In the office, he was found to be orthostatic while standing with a systolic blood pressure of 90 mmHg and a mildly elevated troponin without EKG changes. The patient was then admitted to the hospital on the basis of these abnormalities. His vital signs on admission were unremarkable: he was afebrile with a blood pressure of 119/72 mmHg in sitting position, heart rate of 78 beats per minute, respiratory rate of 16 breaths per minute, and an oxygen saturation of 97% on room air. He underwent a transthoracic echocardiogram, which visualized a freely mobile mass in the right ventricle and atrium. A trans-esophageal echocardiogram demonstrated a 4×4 cm blood clot in the right ventricle with extension into the right atrium and protruding through into the left atrium via a patent foramen ovale (see Video). In light of these findings, a CT angiogram of the chest and a lower extremity venous duplex ultrasound were performed. CT angiogram revealed bilateral pulmonary emboli with heavy clot burden and evidence of right heart strain (see Fig. 1), while the ultrasound described a left-sided venous thrombosis involving both the lesser saphenous and popliteal veins. Intravenous heparin was started.

**Fig. 1:**
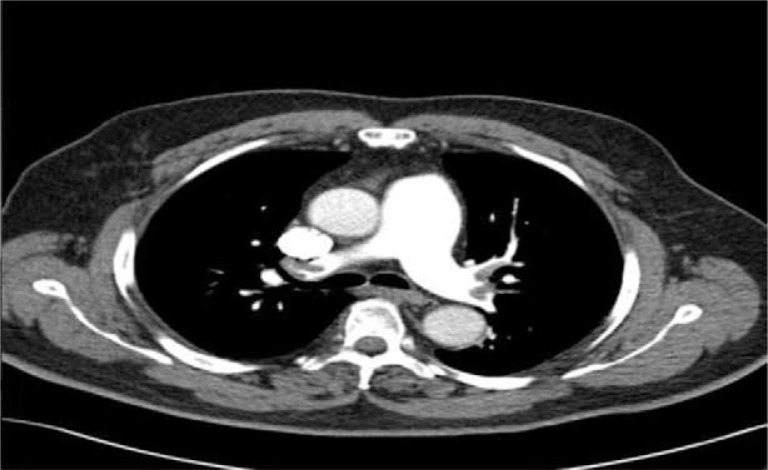
Spiral CT-scan of chest demonstrating large clot burden in the pulmonary arteries bilaterally.

After a detailed discussion with the patient and his family about the options for treatment and their associated prognosis, he declined other interventions including intravenous thrombolytic therapy and surgical thrombectomy. The hospital course was uncomplicated until day 5, when he was noted to have one episode of gross hematuria that resolved without intervention. Urine cytology and urogenital imaging were normal. He later refused a cystoscopy but was up-to-date on all other routine cancer screening recommendations. On hospital day 9, after undergoing a follow-up transthoracic echo consistent with decreased clot burden, the patient was discharged home on oral warfarin therapy. Nine months later, he was feeling well and returned for a follow-up trans-esophageal echocardiogram, which demonstrated complete resolution of the thrombi and a patent foramen ovale (see Video).

**Video:**
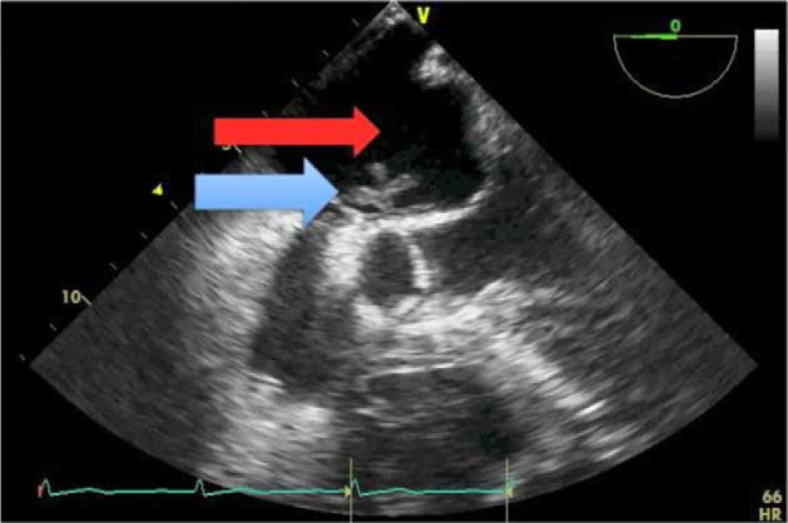
Free floating clot (blue arrow) in left atrium (red arrow).

From a pathophysiological perspective, mobile right heart thrombi (MRHT) are often referred to as ‘in-transit’ or clots traveling from the legs to the pulmonary circuit, and can therefore be categorized as a type of venous thromboembolic disease (1, 2). In our patient, we theorize that the mobile heart thrombus originated from the deep venous thrombosis in the left leg, which likely formed as a result of prolonged immobility from his gastrointestinal illness combined with an obese body habitus. Even in this context, the incidence of mobile right heart thrombi remains a rare occurrence with an estimated incidence of 3.8% in patients with pulmonary embolism, which occurs concomitantly in almost 100% of cases (1, 2). In such patients, the mortality rate from pulmonary embolism is significantly higher than those without right heart thrombi formation (>40%), making rapid diagnosis and treatment critical elements to improving survival (3, 4). While there is agreement regarding use of echocardiography as the most accurate means for diagnosing MRHT (5), no such consensus exists for the most effective management strategy.

At present, the treatment options include thrombolytic therapy, intravenous anticoagulation therapy, and surgical thrombectomy, the outcomes of which have only been compared almost exclusively by retrospective analysis and never by randomized trial. The only prospective case series to date, conducted by Pierre-Justin et al., concluded that intravenous thrombolysis with rt-PA constitutes a safe and efficient first-line intervention (6). Pierre-Justin also discussed the potential risks surrounding clot lysis, including migration of fragments or recurrence of embolism, often known to occur when the venous thrombus is only partially dissolved (6). Due to the threat of paradoxical embolus, the risk of fragment migration with thrombolysis is particularly problematic in the presence of a patent foramen ovale (2, 7, 8), as described in our case, thus leading some authors to favor surgery in such situations (9). In particular, the ability to repair the heart defect while simultaneously removing the intracardiac thrombus presents a key advantage to choosing a surgical approach in patients with a patent foramen ovale (PFO) (2). In contrast, Fauveau et al.'s literature review identified 88 cases of thrombus straddling a patent ovale and proposed that, in cases when the patient was hemodynamically stable and the thrombus itself was small (∼4 cm), successful management could be achieved using anticoagulants alone (9). Fauveau's review also measured the mortality rates between the three treatment strategies and reported a mortality rate of 13% for surgical thrombectomy, 14% for heparin alone, and 36% for thrombolysis (7). It is worth noting however, that the aforementioned mortality rates reflect the outcomes only in cases of thrombus straddling a patent formen ovale. Interestingly, when the literature review was expanded (*n*=177) to include *all* documented cases of right heart thromboemboli, as in the retrospective case series published by Rose et al., the numbers change drastically (2). Even after using multivariate modeling to account for confounding variables, Rose reported a mortality rate of 11.3% for thrombolysis, 23.8% for surgical thrombectomy, and 28.6% for heparin alone, ultimately concluding that thrombolytic therapy conferred a protective effect compared with anticoagulation alone (2). Additionally, at least two other studies have concluded that no significant mortality difference exists between the three regimens (1, 3).

Thus, given the conflicting data available on the management of intracardiac thrombus, it is clear that our case illustrates both an unusual clinical scenario and a therapeutic dilemma which, at present, remains unresolved. A large degree of this controversy likely stems from the inability to determine whether the existing data are skewed due to factors that are outside the control of a non-randomized study, such as varying degrees of selection and reporting bias. For example, authors who reported a high mortality associated with thrombolysis suggested that this finding could be secondary to the severity of the patient's initial presentation, rather than the treatment they received (7). However, authors who reported a more favorable mortality rate with thrombolysis proposed that the patients who received alternative therapies (anticoagulation or surgery) were inherently sicker than their counterparts (2). Ultimately, intracardiac thrombus carries a high risk of mortality and we await more definitive guidance for the optimal treatment of this rare condition, which will likely be answered only by way of a randomized clinical trial.
